# Accurate Deep Potential Model of Temperature-Dependent Elastic Constants for Phosphorus-Doped Silicon

**DOI:** 10.3390/nano15100769

**Published:** 2025-05-20

**Authors:** Miao Gao, Xiaorui Bie, Yi Wang, Yuhang Li, Zhaoyang Zhai, Haoqi Lyu, Xudong Zou

**Affiliations:** 1 The State Key Laboratory of Transducer Technology, Aerospace Information Research Institute, Chinese Academy of Sciences, Beijing 100190, China; gaomiao23@mails.ucas.ac.cn (M.G.); biexr@aircas.ac.cn (X.B.); wangyi235@mails.ucas.ac.cn (Y.W.); liyuhang211@mails.ucas.ac.cn (Y.L.); zhaizhaoyang20@mails.ucas.ac.cn (Z.Z.); lvhaoqi19@mails.ucas.ac.cn (H.L.); 2 School of Electronic, Electrical and Communication Engineering, University of Chinese Academy of Sciences, Beijing 100049, China; 3 QiLu Aerospace Information Research Institute, Jinan 250101, China

**Keywords:** density functional theory, deep potential, molecular dynamics, elastic constants, temperature dependence

## Abstract

Accurate predictions of elastic properties under varying doping concentrations and temperatures are critical for designing reliable silicon-based micro-/nano-electro-mechanical systems (MEMS/NEMS). Empirical potentials typically lack accuracy for elastic predictions, whereas density functional theory (DFT) calculations are precise but computationally expensive. In this study, we developed a highly accurate and efficient machine learning-based Deep Potential (DP) model to predict the elastic constants of phosphorus-doped silicon (Si_64−x_P_x_, x = 0, 1, 2, 3, 4) within a temperature range of 0–500 K. The DP model was rigorously validated against benchmark DFT results. At 0 K, the elastic constants predicted by our DP model exhibited excellent agreement with experimental data, achieving a mean absolute percentage error (MAPE) of only 2.88%. We investigated the effects of doping on elastic constants in single-crystal silicon and determined their second-order temperature coefficients. The calculations demonstrated distinct doping-induced variations, showing pronounced decreases in C_11_ and C_44_ and a moderate increase in C_12_. Finite-element analyses using the fitted temperature coefficients indicated improved thermal stability of silicon resonators through phosphorus doping. Our study explores the integration of machine learning-based atomic-scale simulations with MEMS/NEMS design, providing practical guidance for optimal dopant selection to enhance silicon resonator thermal stability.

## 1. Introduction

Silicon has been widely utilized as the base material for MEMS/NEMS applications, owing to its excellent mechanical properties, well-established fabrication processes, and reliable integration and packaging technologies [1]. MEMS/NEMS silicon resonators exhibit excellent mechanical resonance properties, enabling their extensive application in inertial sensors such as accelerometers, gyroscopes, and frequency reference oscillators [2]. In these high-precision sensing and timing applications, maintaining resonant frequency stability is critical, as frequency drift directly compromises device accuracy and performance [3,4]. However, achieving stable frequencies is challenging due to temperature-induced frequency drift, particularly since silicon typically exhibits a temperature coefficient of frequency (TCF) of approximately −30 ppm/°C [5].

Recent research has revealed that heavy doping in silicon can effectively tailor its elastic constants, providing a viable strategy to mitigate temperature-induced frequency drift in MEMS/NEMS silicon resonators [6,7]. A possible mechanism is that heavy doping introduces strain into the crystal lattice and alters the electronic band structure. This modification causes charge carriers to redistribute in a way that minimizes the free energy of the system, ultimately affecting its elastic properties [8,9,10,11,12]. Therefore, accurately capturing these underlying mechanisms at the atomic scale is essential to elucidate the fundamental relationship between doping effects and elastic properties, enabling more precise design and optimization of MEMS/NEMS silicon resonators.

Atomic-scale simulations are pivotal in multiscale materials modeling, offering both microscopic insights and quantitative inputs for higher-scale models [13,14]. Atomistic simulations are commonly performed using quantum-mechanical methods such as DFT, renowned for their high accuracy, flexibility, and reproducibility [15]. However, due to their high computational cost, DFT-based methods are inefficient for studying large systems, especially under finite-temperature conditions [16]. Empirical force fields (FFs), in contrast, approximate atomic interactions through predefined analytical forms parameterized against experimental or theoretical data [17]. While offering high computational efficiency, these empirical FFs often suffer from limited accuracy and poor transferability, particularly in chemically complex or doped systems [18]. Among them, the Tersoff [19,20,21] and Stillinger–Weber [22] potentials are two of the most widely used models, originally formulated for covalently bonded materials such as silicon and carbon [23]. These limitations motivate the adoption of more accurate approaches such as ab initio molecular dynamics (AIMD), in which interatomic forces are computed directly from first-principles electronic structure calculations (typically DFT) at every MD timestep [24]. AIMD offers DFT-level accuracy and is capable of capturing electronic effects and anharmonic interactions with high fidelity [25]. However, its computational cost is substantially higher than that of classical MD, restricting its application to relatively small systems and short simulation times—typically a few hundred atoms over a few tens of picoseconds [26].

In recent years, machine learning interatomic potentials (MLIPs) have emerged as a transformative tool for bridging the accuracy of quantum mechanical methods and the efficiency of classical force fields [27,28]. Studies have shown that MLIPs offer significant improvements over traditional empirical potentials, typically achieving near-DFT accuracy in energy and force predictions across diverse chemistries and atomic configurations [29]. These advances, along with the remaining challenges in generalization, benchmarking, and data quality, have been comprehensively reviewed by Ko and Ong [30]. Representative models include neural network potentials (NNP) [31], PreFerred Potential (PFP) [32], Gaussian approximation potentials (GAP) [33], spectral neighbor analysis potential (SNAP) [34], atomic cluster expansion (ACE) [35], and moment tensor potentials (MTP) [36], among others [37,38,39]. Among these, the Deep Potential Molecular Dynamics (DPMD) framework stands out for its scalability and robustness. By leveraging deep neural networks trained on extensive DFT datasets, DPMD enables efficient molecular dynamics simulations with DFT-level accuracy, making it particularly suitable for modeling large, complex systems at finite temperatures. Compared to AIMD, this approach significantly improves computational efficiency, facilitating accurate and efficient molecular dynamics simulations and material property predictions. However, the performance of DPMD strongly depends on the comprehensiveness of its initial training dataset, motivating the development of the Deep Potential GENerator (DP-GEN) method [40]. The DP-GEN method is built upon the DPMD framework, employing an active learning strategy to automatically identify critical configurations from molecular dynamics simulations for accurate labeling and iterative training [41]. This approach efficiently and autonomously generates high-accuracy potential models, significantly reducing computational costs and enhancing the generalization capabilities of DP models [42]. DP-GEN has been extensively applied across various material systems, including metals, alloys, and aqueous solutions, demonstrating substantial potential within the field of materials simulation [43,44,45].

In this work, we successfully developed an accurate potential model for Si_64−x_P_x_ (x = 0, 1, 2, 3, 4) using the DP-GEN method, with training data obtained from DFT calculations. The accuracy of the DP model was validated through comparisons with experimental data and first-principles calculations. Using this model, we systematically explored how the doping concentration affects the temperature dependence of elastic constants within a temperature range of 0–500 K. Additionally, we employed finite element simulations to validate the enhancement of thermal stability in the material induced by phosphorus doping. We propose a computational approach to investigate the possibility of the doping-mediated tuning of silicon’s elastic constants, aiming to contribute to improved thermal stability in MEMS/NEMS resonator devices.

## 2. Materials and Methods

### 2.1. Generation of DP Model

**Figure 1:**
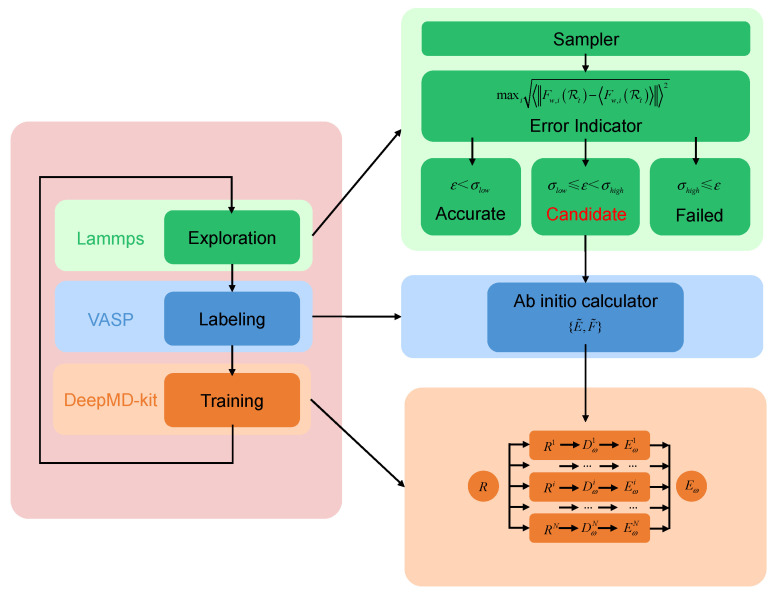
Schematic illustration of the DP-GEN scheme. Green modules represent the sampling and screening processes. The blue module denotes accurate labeling of data via ab initio calculations. The orange module indicates the training process of the DP.

Constructing an accurate and reliable DP model requires the careful selection of training datasets that fully capture the complexity of the system’s potential energy surface. DP-GEN integrates DP models with a parallel active-learning framework to automatically generate high-quality training data, effectively overcoming traditional approaches that typically require extensive manual intervention and substantial computational resources, thereby enhancing both efficiency and accuracy [46]. The core concept of DP-GEN involves three iterative steps—exploration, labelling, and training [47]. In the exploration step, molecular dynamics simulations are performed using LAMMPS (version 17 Apr 2024) [48] to generate new system configurations. During the labelling step, accurate potential energy data are obtained by performing first-principles calculations on these configurations with VASP (version 6.3.2) [49,50]. In the training step, the labelled data are utilized by DeePMD-kit (version 2.2.10) [51] to train and optimize the DP model. Through this iterative workflow, the accuracy and generalization capabilities of the DP model are progressively enhanced. A schematic representation of this iterative process is provided in Figure 1.

In the exploration step, the sampler evolves the initial configuration of the system into a series of new configurations. For a given configuration $R_{t}$, model deviation $\epsilon_{t}$ is defined as the maximum standard deviation of atomic forces predicted by the ensemble of models.

(1) $$\epsilon_{t}=\underset{i}{\max}\sqrt{{\langle \left\|F_{w,i}\left(R_{t}\right)-\langle F_{w,i}\left(R_{t}\right)\rangle \right\|\rangle }^{2}}$$

where $F_{w,i}\left(R_{t}\right)$ denotes the force on the atom with index i predicted by model $E_{w}$, and $\langle F_{w,i}\left(R_{t}\right)\rangle$ denote the expectation with respect to the ensemble of models and are estimated by the average of model predictions. Based on the calculated model deviations, all configurations are categorized into three classes: (1) failed configurations $\left(\sigma_{high}\leq \epsilon_{t}\right)$, which are excluded from the training dataset; (2) candidate configurations $\;(\sigma_{low}\leq \epsilon_{t}<\sigma_{high})$, which can be incorporated into the next iteration only after DFT calculations; and (3) accurate configurations $\left(\epsilon_{t}<\sigma_{low}\right)$, which require no further processing. The lower $\left(\sigma_{low}\right)$ and upper $\left(\sigma_{high}\right)$ force deviation thresholds for screening configurations are set to 0.05 eV/Å and 0.4 eV/Å, respectively.

In the labeling step, the ab initio calculator performs first-principles calculations to obtain the energies ($\tilde{E}$) and forces ($\tilde{F}$) for candidate configurations. The total potential energy E of a configuration is assumed to be the sum of atomic energies $E_{i}$ from each atom i, which are mapped from atomic descriptor $D_{i}$ through an embedding network [52]. Descriptor $D_{i}$ characterizes the local environment of atom i within cutoff radius $R_{c}$, set as 6 Å. The maximum number of atoms within $R_{c}$ is fixed at 50.

In the training step, the embedding network consists of multilayer fully connected layers containing 25, 50, and 100 neurons, respectively, mapping the atomic local environment descriptors into atomic energies. The fitting network comprises three fully connected layers, each with 240 neurons, designed to accurately predict the total potential energy, atomic forces, and the virial tensor of the system. We used the hyperbolic tangent (tanh) function as the activation function and trained the model using the Adam optimizer with an exponentially decaying learning rate. This fitting network is trained using a residual neural network architecture. The loss function quantifies the difference between the predicted energy, atomic forces, and virial tensor from the DP model and the corresponding values in the training dataset, and is defined as follows:

(2) $$L\left(p_{\varepsilon },p_{f},p_{\xi }\right)=p_{\varepsilon }\Delta \epsilon^{2}+\frac{p_{f}}{3N}\sum_{i}{\left|\Delta F_{i}\right|}^{2}+\frac{p_{\xi }}{9}\|\Delta \xi {\|}^{2}$$

Here, Δϵ denotes the error between the predicted and reference energies, $\Delta F_{i}$ represents the difference between the predicted and reference atomic forces, and Δξ is the prediction error of the virial tensor. Parameters $p_{\varepsilon }$, $p_{f}$, and $p_{\xi }$ are weight coefficients assigned to the energy, force, and virial tensor terms within the loss function, respectively, and are used to control their relative contributions to the total loss. We set the training steps to 1,000,000, during which weights $p_{\varepsilon }$ and $p_{\xi }$ are dynamically adjusted from 0.02 to 1, allowing the loss function to progressively optimize throughout the training process.

**Figure 2:**
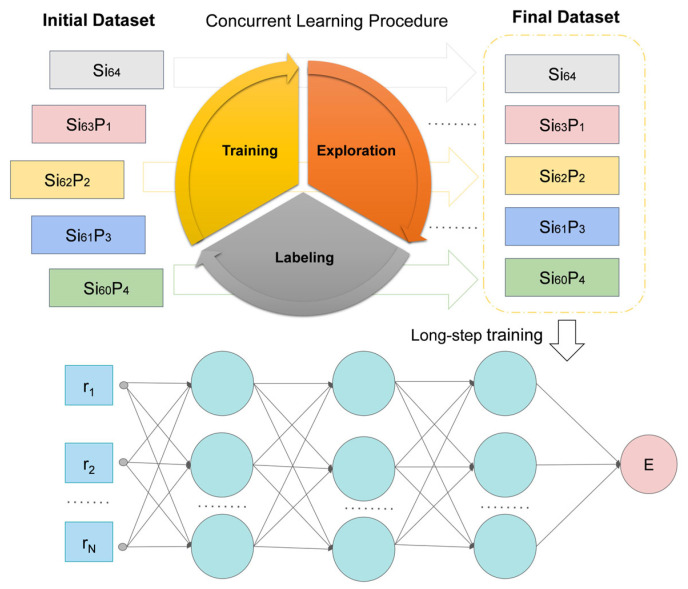
The workflow of the DP model generation for Si_64−x_P_x_. Iterative training with different components individually, including x = 0, 1, 2, 3 and 4. After convergence of each component, the datasets with different *x* are collected into the final dataset. A DP model is generated through a long-step training based on this final training dataset.

We constructed a Si_64_ structure by expanding the conventional silicon cell into a 2 × 2 × 2 supercell. Si_64−x_P_x_ models with continuous doping concentrations (x = 0, 1, 2, 3, 4) were then created by randomly substituting Si atoms with phosphorus atoms. Such doping concentrations were sufficiently high to cover practically relevant doping ranges for MEMS/NEMS applications. Higher doping levels would have introduced strong dopant-dopant interactions, necessitating significantly larger supercells and increased computational costs. Therefore, we chose to stop our calculations at x = 4. The atomic coordinates of these structures were appropriately perturbed to generate initial structural configurations. As shown in Figure 2, during the DP-GEN training process, systems with different doping concentrations (x) were iteratively trained separately. After convergence of each individual system, all collected data were integrated into a unified dataset for an extended training procedure. This training approach effectively describes system properties across continuously varying doping concentrations, achieving accuracy comparable to the individually trained models specific to each doping concentration [53]. Moreover, this approach significantly reduces the required computational resources, thus markedly enhancing overall computational efficiency [54].

### 2.2. DFT and MD Simulation Settings

DP-GEN performs DFT single-point calculations on candidate configurations intended for inclusion in the dataset, further optimizing and evaluating the system’s energy and structural properties. All DFT calculations were performed using the first-principles software VASP to ensure accuracy and consistency. The exchange–correlation functional was treated using the generalized gradient approximation (GGA), specifically employing the Perdew–Burke–Ernzerhof (PBE) functional [55]. The ion-electron interaction was described using the projector augmented wave (PAW) method [56]. Based on thorough testing, a cutoff energy of 500 eV was adopted for the plane-wave basis across all calculations, with a KSPACING value of 0.5. The energy convergence criterion for electronic self-consistency was set at a stringent threshold of 1.0 × 10^−6^ eV per atom. For each structure, a maximum of 60 candidate configurations and a minimum of 10 were calibrated. Default recommended parameters were used for all other settings to maintain accuracy while avoiding excessive computational overhead. The phonon spectrum was computed using the Phonopy package for model validation. The force constant matrix was generated via the finite displacement method, and a 2 × 2 × 2 supercell was employed for the simulations [57].

At the exploration step, MD simulations were performed using the LAMMPS package, with interatomic interactions described by intermediate DP models generated during the iterative training process of the DP-GEN workflow. The simulations were conducted in the isothermal–isobaric (NPT) ensemble, with the temperature ranging from 0 to 500 K, the pressure ranging from 1 to 10 kbar, and simulation times ranging from 1 to 50 ps. The time step was set to 0.001 ps. More details of the exploration strategy can be found in Table 1. The time step for the MD simulation was set to 1 fs. The temperature and pressure were controlled using the Nose–Hoover thermostat and the Parrinello–Rahman barostat, respectively.

### 2.3. Elastic Constants Calculation

As a typical cubic crystal, silicon has an elastic stiffness tensor that can be simplified into a matrix containing only three independent constants (C11, C12, C44):

(3) $$\left[\begin{array}{c} \sigma_{1}\\ \sigma_{2}\\ \sigma_{3}\\ \sigma_{4}\\ \sigma_{5}\\ \sigma_{6} \end{array}\right]=\left[\begin{array}{cccccc} C_{11} & C_{12} & C_{12} & 0 & 0 & 0\\ C_{12} & C_{11} & C_{12} & 0 & 0 & 0\\ C_{12} & C_{12} & C_{11} & 0 & 0 & 0\\ 0 & 0 & 0 & C_{44} & 0 & 0\\ 0 & 0 & 0 & 0 & C_{44} & 0\\ 0 & 0 & 0 & 0 & 0 & C_{44} \end{array}\right]\left[\begin{array}{l} \epsilon_{1}\\ \epsilon_{2}\\ \epsilon_{3}\\ \epsilon_{4}\\ \epsilon_{5}\\ \epsilon_{6} \end{array}\right]$$

The stress–strain method is widely utilized for computing elastic constants by applying small strains to a material’s equilibrium structure and determining the elastic constants from the resulting stress–strain relationships. Nevertheless, this approach is limited in its capability to accurately capture the temperature dependence of elastic constants. Squire et al. showed that stress tensor σ is the first derivative of free energy with respect to strain, while elastic tensor C corresponds to the second derivative of free energy with respect to strain [58]. Additional derivations and details can be found in the studies by Lutsko [59] and Van Workum et al. [60].

The stress tensor is:

(4) $$\sigma_{\alpha \beta }=\sigma_{\alpha \beta }^{B}-\rho k_{B}T\delta_{\alpha \beta }$$

The elastic stiffness tensor is:

(5) $$C_{\alpha \beta \mu \nu }=\langle C_{\alpha \beta \mu \nu }^{B}\rangle -\frac{V}{k_{B}T}\left[\langle \sigma_{\alpha \beta }^{B}\sigma_{\mu \nu }^{B}\rangle -\langle \sigma_{\alpha \beta }^{B}\rangle \langle \sigma_{\mu \nu }^{B}\rangle \right]+\frac{Nk_{B}T}{V}\left(\delta_{\alpha \mu }\delta_{\beta \nu }+\delta_{\alpha \nu }\delta_{\beta \mu }\right)$$

where

(6) $$C_{\alpha \beta \mu \nu }^{B}=\frac{1}{V}\frac{\partial^{2}U}{\partial \epsilon_{\alpha \beta }\partial \epsilon_{\mu \nu }}$$

and

(7) $$\sigma_{\alpha \beta }^{B}=\frac{1}{V}\frac{\partial U}{\partial \epsilon_{\alpha \beta }}$$

Here, the total potential energy of the system is U, T is the temperature, V is the volume, N is the number of particles, $k_{B}$ is Boltzmann’s constant, and $\delta_{\alpha \beta }$ is the Kronecker identity tensor [61]. The first term in Equation (5), known as the Born term, represents the configurational contribution to the elastic stiffness tensor. The second term, $\sigma_{\alpha \beta }^{B}$, represents the Born contribution to the stress tensor, which is why this method is also known as the stress-fluctuation method [62]. The third term corresponds to the ideal gas contribution, associated with the strain derivatives of the volume. Generally, only the first two terms have a significant impact on the elastic constants [63].

To compute the elastic constants at finite temperatures, we performed MD simulations using the final DP model trained via the DP-GEN workflow on a DFT-labeled dataset containing diverse phosphorus-doped silicon configurations. The MD simulations were carried out in LAMMPS, interfaced with the DeepMD-kit, using the converged DP model obtained from long-time iterative training. We utilized the compute born/matrix command in LAMMPS, which calculates the Born matrix to evaluate the elastic constants based on statistical averages of stress fluctuations under thermodynamic equilibrium. Compared to traditional static stress-strain methods, our approach greatly improved the accuracy of elastic constant calculations by utilizing multiple thermodynamic equilibrium samplings and precise evaluations of stress fluctuations. However, accurately sampling stress fluctuations was also the most computationally expensive part of the process.

## 3. Results and Discussion

### 3.1. Accuracy of DP Model

**Figure 3:**
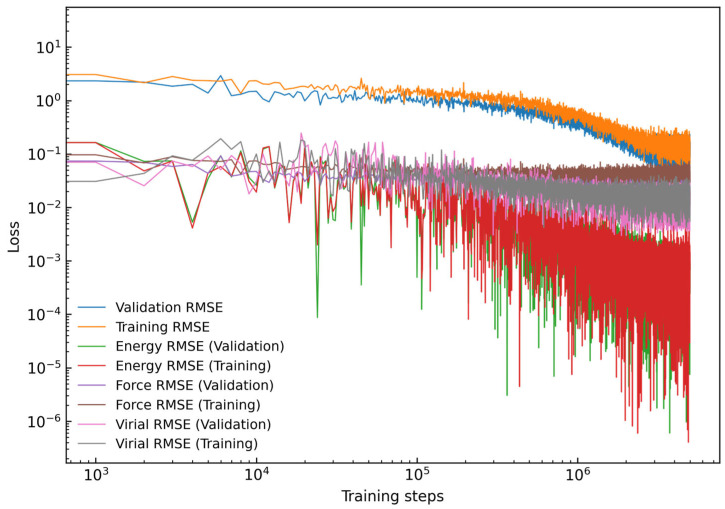
Evolution of root mean square error (RMSE) for training and validation datasets over training steps. The plotted parameters include total RMSE, energy, force and virial terms.

After the DP-GEN workflow had finished, a representative set of training data was generated. A final high-performing DP model was trained over 5,000,000 steps, with the learning rate exponentially decaying from 1.0 × 10^−3^ to 3.5 × 10^−8^. The loss function curves for energy and force during the training process are presented in Figure 3. The energy error was measured in units of eV, while the force error was expressed in eV/Å [64]. The labels in the legend, from top to bottom, represent the total RMSE, energy RMSE, and atomic force RMSE in the test and training sets. As the number of training steps increased, the model’s energy learning curve exhibited a progressively decreasing trend. By step 10^6^, the curve flattened and stabilized around a consistent error value, clearly indicating the model’s convergence.

**Figure 4:**
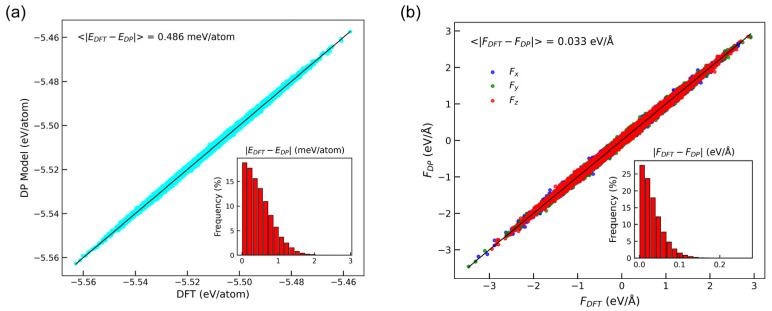
Comparative analysis of (**a**) total energies and (**b**) atomic forces for Si_64−x_P_x_ systems with varying phosphorus compositions (x = 0, 1, 2, 3, 4), obtained from DP predictions versus DFT calculations.

To validate the accuracy of the DP model, we compared the energies and forces predicted by the DP model with the reference data obtained from AIMD calculations performed on Si_64−x_P_x_ systems with varying compositions (x = 0, 1, 2, 3, 4). As shown in Figure 4, the data points for both energies and forces lay closely along the line of y = x. The mean absolute errors between DP and DFT for energies and atomic forces were 0.486 meV/atom and 0.033 eV/Å, respectively. These values fell well within the range commonly considered highly accurate for MLIPs, as recent studies have reported average errors as low as 1 meV/atom for energies and 0.05 eV/Å for atomic forces in conventional MLIP benchmarks [65]. This demonstrates that the developed DP model provides reliable predictions for both energies and forces.

**Figure 5:**
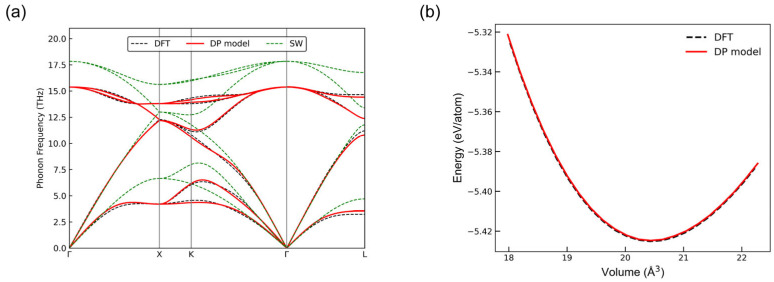
Comparison of the DP model predictions and DFT results for monocrystalline silicon: (**a**) phonon spectra and (**b**) equation of state.

In addition, to comprehensively evaluate the accuracy of the DP model in describing material properties, we performed extensive validations, including calculations of phonon spectra, EOS, and elastic constants. Figure 5 illustrates the phonon dispersion curves for silicon calculated along various high-symmetry directions in the Brillouin zone, comparing results obtained from DFT calculations and the empirical Stillinger-Weber (SW) potential. The results from the DP model exhibited excellent overall agreement with those from DFT, accurately capturing all intersection points of the phonon dispersion curves. The EOS describes the energy variation of a material as a function of volume and serves as a critical indicator for assessing the accuracy of potential energy models. Our calculated EOS curve for silicon closely matched the results obtained from DFT calculations, indicating that our DP model accurately captured the equilibrium structure of silicon.

**Figure 6:**
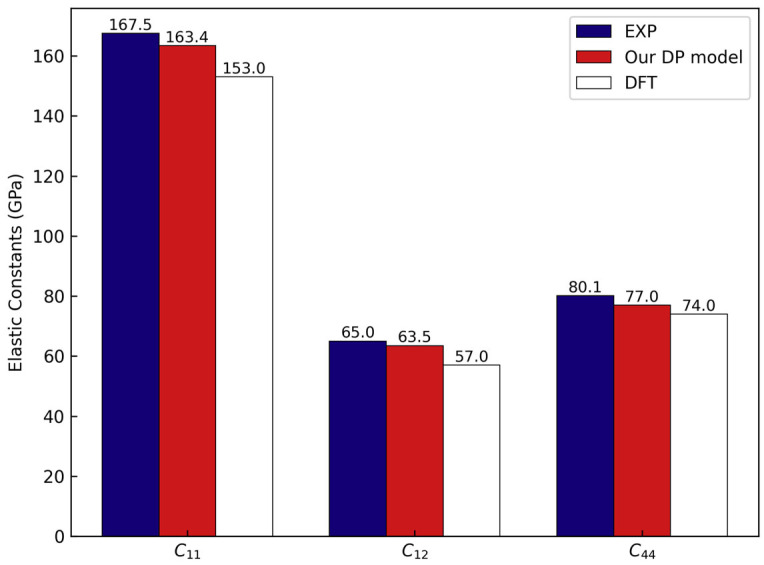
Comparison of elastic constants obtained from experimental measurements [66], our DP model, and reference DFT calculations [67].

We focused specifically on evaluating the accuracy of the DP model in describing the elastic properties of materials. The elastic constants of silicon at 0 K were calculated, and comparisons are presented with experimental data [66] and results from previous studies [67]. As illustrated in Figure 6, our calculated elastic constants C11, C12, and C44 exhibited errors of −2.45%, −2.31%, and −3.87%, respectively, relative to experimental values, resulting in a mean absolute percentage error (MAPE) of 2.88%. The reference DFT calculations employed the GGA-PBE functional, identical to our DP-GEN model training, and our DP model provided even more accurate elastic constant predictions.

These results demonstrate that the single DP model developed in this study accurately described the high-dimensional potential energy surface of silicon systems across varying phosphorus doping concentrations. This highlights the exceptional generalization capability and flexibility of the DP model in modeling complex doped systems.

### 3.2. Dopant Concentration Dependence of Elastic Constants

**Figure 7:**
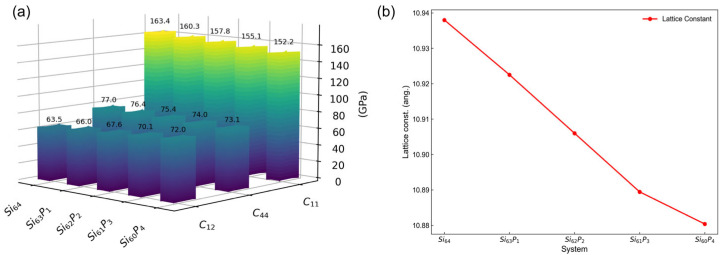
Relationship between doping concentration and (**a**) elastic constants, and (**b**) lattice constants at 0 K.

In this section, we employed the neural-network-based DP model to systematically explore how the dopant concentration affected the elastic constants of silicon. Figure 7a illustrates the variation in elastic constants (C_11_, C_12_, and C_44_) as a function of phosphorus doping concentration at 0 K. Our results clearly indicate that increasing phosphorus doping reduced the elastic constants C_11_ and C_44_, while it concurrently increased C_12_. Additionally, by comparing the elastic constants of pure Si (Si_64_) and phosphorus-doped Si (e.g., Si_60_P_4_), we found that with increasing doping concentration, the variations in C_11_ and C_44_ were more pronounced than those in C_12_. This indicates that C_11_ and C exhibited greater sensitivity to phosphorus doping concentration compared to C_12_.

The doping-dependent variations observed in the elastic constants of silicon can be understood by considering the combined effects of lattice distortion and electronic redistribution induced by phosphorus doping [68]. Specifically, phosphorus atoms, having a smaller atomic radius than silicon atoms, introduce lattice strain, resulting in lattice contraction as evidenced by the reduction of lattice constants shown in Figure 7b. Such lattice strain modifies the electronic band structure, prompting a redistribution of charge carriers that minimizes the overall free energy of the system. As phosphorus is an n-type dopant, it significantly enhances the free-electron concentration, further altering the electronic environment within the silicon lattice. According to Keyes’ theory [9], the electronic contributions to the elastic constants can be expressed as:

(8) $$\Delta C_{11}=-\frac{2N\Xi_{u}^{2}}{9E_{f}}\frac{F_{1/2}^{\prime }(\eta )}{F_{1/2}(\eta )}\;\Delta C_{12}=\frac{N\Xi_{u}^{2}}{9E_{f}}\frac{F_{1/2}^{\prime }(\eta )}{F_{1/2}(\eta )}\;\Delta C_{44}=0$$

where N is the electron concentration, $\Xi_{u}$ is the uniaxial deformation potential constant, F is the Fermi integral, η is $E_{f}/kT$, and $E_{f}$ is the Fermi energy.

From the above equations, it is clear that the coefficient for ΔC_11_ is negative, while the coefficient for ΔC_12_ is positive. This directly explains why an increase in doping concentration resulted in a decrease of C_11_ and an increase of C_12_. Indeed, Keyes’ theory, which exclusively considers electronic redistribution effects of free carriers, predicts that the elastic constant C_44_ will remain unchanged upon doping. Nevertheless, our findings indicate other effects, such as dopant-induced lattice distortions, causing measurable changes in C_44_. This prediction aligns well with experimental measurements reported by Jaakkola et al. [2], who observed a clear decrease in C_44_ (from 79.2 GPa to 78.5 GPa) as phosphorus doping concentration increased from 4.1 × 10^19^ cm^−3^ to 7.5 × 10^19^ cm^−3^.

Consequently, these structural and electronic modifications strongly influenced elastic constants associated with longitudinal (C_11_) and shear (C_44_) deformation modes, leading to more pronounced changes in these parameters. Conversely, the relatively minor variation observed in C_12_ implied that transverse or coupling deformation modes were less sensitive to phosphorus-induced lattice strain and electronic redistribution. This anisotropic response highlights the selective sensitivity of silicon’s elastic properties to doping concentration.

### 3.3. Temperature Dependence of Elastic Constants

**Figure 8:**
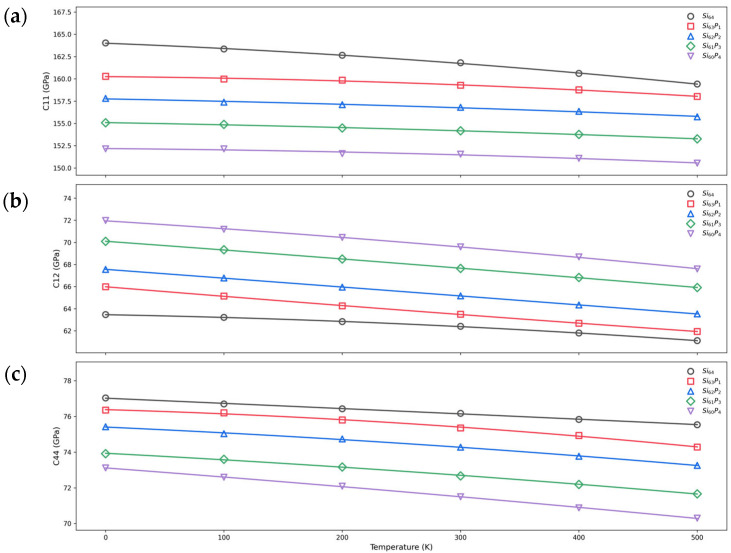
Elastic constants (C_11_, C_12_, and C_44_) for doping concentrations x = 0, 1, 2, 3, and 4 over the temperature range of 0–500 K: (**a**) C_11_, (**b**) C_12_, and (**c**) C_44_.

In this section, we utilized the Born-matrix method implemented in LAMMPS to calculate the elastic constants at finite temperatures, systematically exploring their temperature dependence. Calculations were carried out over a temperature range extending from 0 K to 500 K, with intervals of 100 K, to examine the detailed evolution of the elastic properties. Figure 8 illustrates the obtained temperature dependence, clearly showing that all elastic constants generally decreased with increasing temperature. This behavior was consistent with thermal softening, where increased temperature enhances atomic vibrations, resulting in greater lattice anharmonicity [69,70]. Such enhanced lattice vibrations effectively weakened the interatomic bonding, reducing the stiffness of the material and thereby decreasing the magnitude of the elastic tensor elements. Similar trends of temperature-induced elastic properties reduction have also been reported previously in the literature [71], confirming the consistency and reliability of our computational results.

For a closer investigation of the temperature dependency of the $C_{ij}$ curves, second-order polynomials centered at $T_{0}=25$ °C were fitted to the elastic parameter data as

(9) $$C_{ij}=C_{ij}^{0}\left[1+a_{ij}\left(T-T_{0}\right)+b_{ij}{\left(T-T_{0}\right)}^{2}\right]$$

where $a_{ij}$ and $b_{ij}$ are the first-order and second-order temperature coefficients, respectively, and $C_{ij}^{0}$ is the constant term; $C_{ij}$ is the elastic constant at temperature T.

The temperature sensitivity of elastic constants was significantly influenced by doping. As indicated by the fitted temperature coefficients presented in Table 2, the absolute values of the first-order temperature coefficients ($a_{11}$) of elastic constant $C_{11}$ decreased notably with increasing phosphorus doping concentration, indicating that doping reduced the temperature dependence of $C_{11}$ in silicon crystals. In contrast, the absolute values of the first-order temperature coefficients ($a_{44}$) for elastic constants $C_{44}$ increased with doping concentration, suggesting enhanced temperature sensitivity for these two elastic constants. This enhancement implied that doping-induced lattice strain and electronic structure modification significantly altered the temperature response of transverse deformation modes.

Regarding second-order temperature coefficients ($b_{ij}$), despite being considerably smaller in magnitude than first-order coefficients, they also exhibited clear doping-concentration dependence. Specifically, doped silicon showed larger absolute values of second-order temperature coefficients for $C_{44}$ compared to pure silicon, while the absolute values for $C_{12}$ and $C_{11}$ in doped silicon were smaller. Moreover, as the doping concentration increased, some second-order coefficients (e.g., $b_{12}$ for Si_63_P_1_) even became positive (21.5 × 10^−3^ ppb/K^2^), indicating the possibility of non-monotonic temperature dependence in elastic constants upon doping.

### 3.4. Temperature Stability of MEMS Resonators

**Figure 9:**
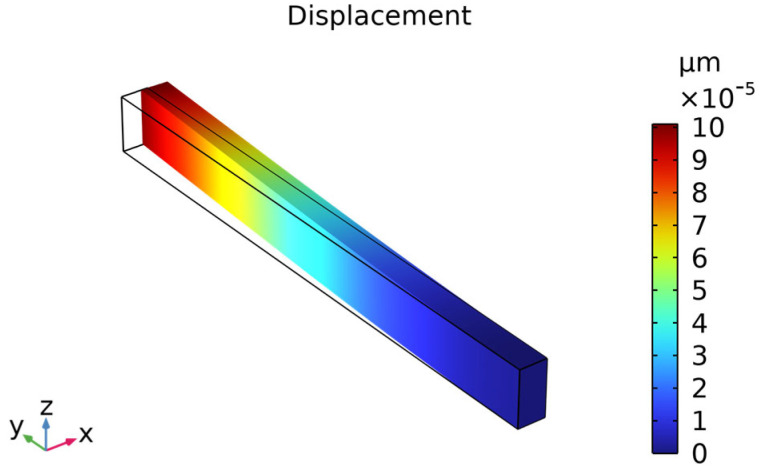
Finite-element model of a cantilever beam illustrating its in-plane vibration modes. The beam dimensions are 100 µm (length), 5 µm (width), and 10 µm (thickness). Color gradient indicates displacement magnitude.

To further investigate the effect of doping on the temperature stability of silicon-based MEMS resonators, we established a finite-element model of microscale cantilever beam using COMSOL Multiphysics (version 6.2) and analyzed its frequency–temperature response characteristics in in-plane vibration modes. The cantilever beam model had dimensions of 100 µm in length, 5 µm in width, and 10 µm in thickness. The elastic parameters of silicon in the model were adopted from our previously fitted experimental results. Through finite-element modal analysis, we obtained the in-plane vibration modes of the cantilever beam along specific crystal orientations (<100> and <110>). Figure 9 presents a representative in-plane vibration mode shape and its corresponding displacement field distribution, where the color gradient indicates displacement magnitude, visually illustrating the deformation characteristics of the cantilever beam under resonance conditions.

Based on the simulation results, we further investigated the dependence of resonance frequency on temperature under various phosphorus doping concentrations. To quantitatively evaluate doping effects on thermal stability, we analyzed the TCF, which directly reflected the frequency sensitivity of MEMS resonators to temperature variations. The TCF is defined as follows:

(10) $$\mathrm{TCF}=\frac{f_{T_{\max}}-f_{T_{\min}}}{\left(T_{\max}-T_{\min}\right)f_{0}}\times 10^{6}$$

where $f_{T_{\max}}$ and $f_{T_{\min}}$ represent the resonance frequencies at the maximum ($T_{\max}$) and minimum ($T_{\min}$) temperatures, respectively, and $f_{0}$ is the resonance frequency at room temperature (25 °C).

**Figure 10:**
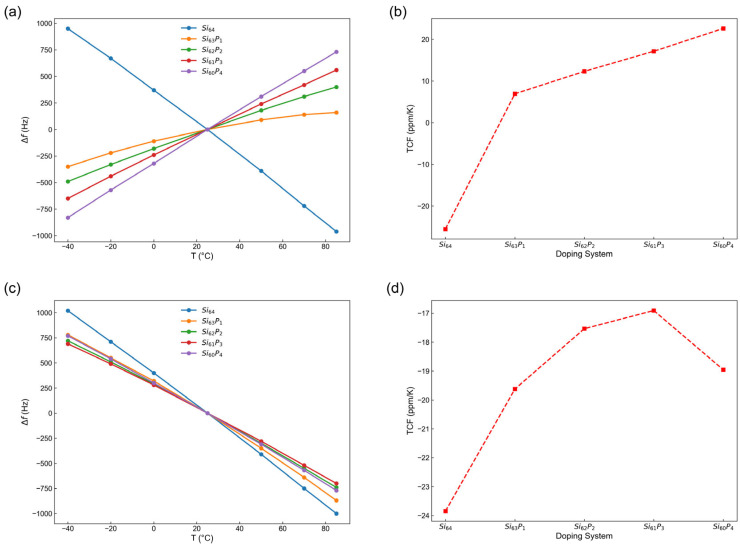
Temperature-dependent frequency variation and TCF for silicon cantilever resonators with various phosphorus doping concentrations oriented along (**a**,**b**) the <100> and (**c**,**d**) the <110> crystallographic directions.

Figure 10a,b illustrate the frequency variation (Δf) versus temperature (T) curves and corresponding TCF curves for silicon cantilever resonators oriented along the <100> direction with different phosphorus doping concentrations. From Figure 10a, it is clearly observed that the resonance frequency of undoped silicon cantilever decreased with increasing temperature, indicating a negative temperature coefficient. However, as the phosphorus doping concentration increased, the frequency variation gradually transitioned to a positive slope, meaning that the resonance frequency increased with rising temperature. Figure 10b further quantitatively demonstrates this trend: as the doping concentration increased, the TCF shifted from negative values to positive values, with the magnitude consistently increasing. This behavior clearly indicates that phosphorus doping effectively modulated and significantly improved the thermal stability of silicon cantilever resonators along the <100> direction, even reversing the sign of the temperature coefficient.

Figure 10c,d present the frequency–temperature responses and TCF curves for silicon cantilever resonators oriented along the <110> direction under different doping concentrations. As shown in Figure 10c, the frequency–temperature response consistently exhibited a negative slope for all doping concentrations, indicating that the resonance frequency decreases steadily with increasing temperature regardless of doping level. Figure 10d further reveals that, while the absolute value of the TCF generally decreased with increased doping, there was a turning point in the doping level of three phosphorus atoms, after which the trend reversed slightly. Excessively increasing the phosphorus doping concentration resulted in a gradual increase in the absolute value of the TCF among the doped models, suggesting the existence of an optimal doping range. Consequently, our findings emphasize the necessity of carefully selecting appropriate doping concentrations to achieve optimal thermal stability.

## 4. Conclusions

In this study, we developed a machine-learning-based DP model to accurately predict the elastic constants of phosphorus-doped silicon at finite temperatures. The accuracy of the DP model was validated through comprehensive comparisons of energies, forces, phonon spectra, and EOS with DFT calculations, exhibiting excellent agreement. At 0 K, the elastic constants predicted by the DP model had a mean absolute percentage error of less than 3% relative to experimental values. Utilizing this validated DP model, we investigated the influence of phosphorus doping concentration on elastic properties, revealing that elastic constants C_11_ and C_44_ consistently decreased with increasing doping concentration, whereas C_12_ exhibited an opposite, increasing trend. Furthermore, the elastic constants were computed within a temperature range of 0–500 K using the Born matrix method, and second-order temperature coefficients were obtained via polynomial fitting. The results indicated that phosphorus doping decreased the temperature sensitivity of C_11_ but increased the temperature sensitivities of C_12_ and C_44_. Finally, finite-element simulations further validated that phosphorus doping significantly enhanced the thermal stability of silicon cantilever beam resonators, underscoring the critical role of selecting appropriate doping concentrations and crystallographic orientations for MEMS/NEMS thermal compensation design. This integrated modeling approach, combining machine-learning-derived DP potentials and finite-element analysis, provides accurate predictions of elastic properties and offers valuable insights for optimizing material selection and enhancing temperature stability in advanced MEMS/NEMS resonator applications.

## Figures and Tables

**Table 1:** Exploration strategy for the phosphorus-doped system. For simulations which adopted the NPT ensemble, 1, 10, 100, 1000, and 10,000 Bar were set as the pressures.

Iter.	Length (ps)	T (K)	Ensemble
0	1	0, 100, 200, 300, 400, 500	NPT
1	1	0, 100, 200, 300, 400, 500	NPT
2	3	0, 100, 200, 300, 400, 500	NPT
3	3	0, 100, 200, 300, 400, 500	NPT
4	5	0, 100, 200, 300, 400, 500	NPT
5	10	0, 100, 200, 300, 400, 500	NPT
6	10	0, 100, 200, 300, 400, 500	NPT
7	20	0, 100, 200, 300, 400, 500	NPT
8	20	0, 100, 200, 300, 400, 500	NPT
9	30	0, 100, 200, 300, 400, 500	NPT
10	30	0, 100, 200, 300, 400, 500	NPT
11	50	0, 100, 200, 300, 400, 500	NPT

**Table 2:** Fitted second-order temperature coefficients of elastic constants for systems with different doping concentrations.

System	$C_{11}^{0}$ (GPa)	$a_{11}$ (ppm/K)	$b_{11}$ (ppb/K^2^)	$C_{12}^{0}$ (GPa)	$a_{12}$ (ppm/K)	$b_{12}$ (ppb/K^2^)	$C_{44}^{0}$ (GPa)	$a_{44}$ (ppm/K)	$b_{44}$ (ppb/K^2^)
Si_64_	161.8	−61.5	−50.1	62.4	−83.8	−88.2	76.2	−39.1	−2.1
Si_63_P_1_	159.3	−31.8	−41.6	63.5	−125.5	21.5	75.4	−61.2	−60.2
Si_62_P_2_	156.8	−26.8	−18.7	65.2	−124.2	−3.8	74.3	−61.4	−35.6
Si_61_P_3_	154.2	−25.4	−18.6	67.7	−125.2	−17.6	72.7	−65.8	−30.8
Si_60_P_4_	151.5	−24.0	−28.5	69.6	−129.5	−54.8	71.5	−81.0	−19.0

## Data Availability

The data that support the findings of this study are available from the corresponding author upon reasonable request.
